# Hydro­chlorides, hydrates, hydro­nitrate, and an unanti­cipated hydrolysis product of famotidine

**DOI:** 10.1107/S2053229626004122

**Published:** 2026-04-24

**Authors:** MacKenzie C. Weaver, Allen G. Oliver, Toni L. O. Barstis

**Affiliations:** a Department of Chemistry and Physics St Mary's College, Notre Dame IN 46556 USA; b Department of Chemistry and Biochemistry, University of Notre Dame, Notre Dame, IN 46556, USA; University of Sydney, Australia

**Keywords:** crystal structure, famotidine, polymorphism, paper-based analytical devices, PADs, active pharmaceutical ingredient, API, GERD, gastroesophageal reflux disease

## Abstract

This article contributes to the development of Paper-Based Analytical Devices (PADs), a low-cost field-friendly platform for screening low-quality medicines. Our investigation focuses on famotidine, the active pharmaceutical ingredient (API) in Pepcid AC [an over-the-counter medicine used to treat gastroesophageal reflux disease (GERD)]. We report the successful isolation and characterization of several new crystalline forms of famotidine, focusing on the API itself rather than its PAD-activated colored com­plex. These forms include a famotidine hydro­chloride polymorph, C_6_H_14_N_7_O_2_S^+^·Cl^−^ (**I**), a famotidine hydro­chloride hemihydrate salt, C_6_H_14_N_7_O_2_S^+^·Cl^−^·0.5H_2_O (**II**), and a famotidine nitrate salt, C_6_H_14_N_7_O_2_S^+^·NO_3_^−^ (**III**). Unexpectedly, we also characterized a hydrolyzed famotidine com­plex, *N*-(di­amino­methyl­ene)-4-({[3-oxo-3-(sul­fam­oyl­amino)­prop­yl]sul­fan­yl}meth­yl)thia­zol-2-am­in­ium chloride ses­qui­hy­drate, C_8_H_15_N_6_O_3_S_3_^+^·Cl^−^·1.5H_2_O (**IV**). The crystal structures reveal significant solid-state diversity: hemihydrate salt **II** exhibits two symmetry-independent famotidine hydro­chloride mol­ecules per asymmetric unit, while sesquihydrate salt **IV** shows four crystallographically-independent hydro­chloride mol­ecules and six symmetry-independent water mol­ecules per standard unit. All four com­plexes display extensive hy­dro­gen-bonded networks in the solid state. The detailed structural characterization of these crystalline com­plexes generates fundamental solid-state chemistry data; this knowledge is essential for predicting and controlling the drug performance and formulation stability of famotidine and crucially informs our development of PADs.

## Abstract 2

The structures of a new polymorph of famotidine hydro­chloride, a famotidine hydro­chloride hydrate, a new hydro­nitrate salt of famotidine, and an unexpected hydrolyzed com­plex of famotidine are reported. The famotidine hydro­chloride hydrate and the hydrolyzed famotidine hydro­chloride have *Z*′ values of 2 and 4, respectively.

## Introduction

The active pharmaceutical ingredient (API) famotidine is a histamine H_2_-receptor antagonist that is available as an over-the-counter (OTC) medicine with the brand name of Pepcid. It is classified as an anti­gastroesophageal reflux disease (anti-GERD) medicine that decreases the amount of gastric acid produced by the stomach (Kapoor *et al.*, 2005 ▸).

We have developed an anti­gastroesophageal reflux disease (anti-GERD) paper-based analytical device (PAD) as a low-cost field-friendly reliable tool that allows untrained users to screen for low-quality medicines (Barstis *et al.*, 2016 ▸), in­clu­ding an anti-GERD PAD to screen for low-quality Pepcid. On these PADs, we have incorporated three key colorimetric tests for the APIs present in various anti-GERD medicines; however, the chemistry of these tests is not well understood. Our research goal is to better understand the chemistry occurring on the anti-GERD PAD by elucidating the chemical structures of the colored famotidine–metal com­plexes *via*X-ray crystallography. We began with a modest study of the chemical structures of the polymorphs, salts, and solvates of the API famotidine, the parent com­pound.

Chemical structures of polymorphic APIs, and their salts, are of inter­est to pharmaceutical manufacturing com­panies, because of potential variation in bioactivities and synthesis efficiencies. Simultaneously, polymorphism in APIs, including famotidine, is a serious concern for pharmaceutical manufacturing com­panies. Formerly, APIs were thought to exist in only one form; however, different polymorphs of these APIs are known to exist. These different polymorphs have varying packing properties, as well as physical properties (*e.g.* melting point, solubility, dissolution rate, and thermal stability), so the full characterization of the APIs, including their crystal structure, must be included as part of the pharmaceutical industry’s drug discovery, development, and optimization processes. One case that illustrates the importance of the full characterization of API polymorphs was the high-profile case of the API Ritonavir (Norvir), an anti­retroviral medicine manufactured by Abbott Laboratories (now AbbVie, Inc.) (Bauer *et al.*, 2001 ▸; Morissette *et al.*, 2003 ▸; Bučar *et al.*, 2015 ▸). An excellent treatise on polymorphism is detailed in Bernstein’s monograph (Bernstein, 2023 ▸).
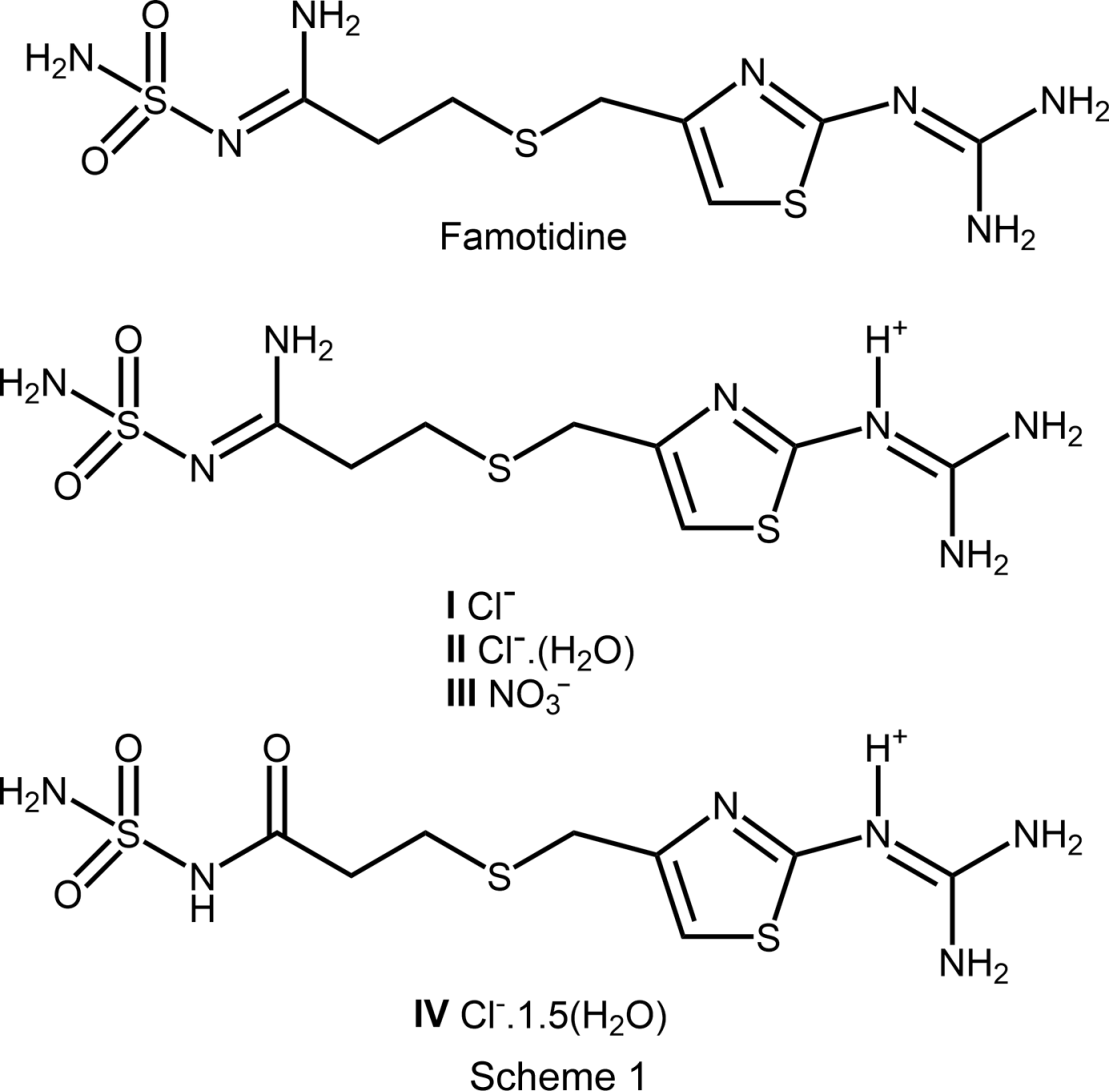


Polymorphism occurs when the same solid material packs in different orientations. Famotidine crystallizes in three forms (Form A, Form B, and Form C) (Yanagisawa *et al.*, 1987 ▸; Golič *et al.*, 1989 ▸; Ferenczy *et al.*, 2000 ▸; Shankland *et al.*, 2002 ▸; Florence *et al.*, 2003 ▸; Overgaard & Hibbs, 2004 ▸; Saikia *et al.*, 2019 ▸), depending on the cooling rates and solvents used (Hassan *et al.*, 1997 ▸; Lu *et al.*, 2007*a* ▸; Lu *et al.*, 2007*b* ▸; Takebayashi *et al.*, 2021 ▸; Soto & Svärd, 2021 ▸), but Form A and Form B are the most commonly discussed polymorphs in the literature. Form C is a metastable form that has not been structurally characterized. Powder diffraction patterns of this form display broad low-intensity peaks indicative of nanocrystalline material (Hassan *et al.*, 1997 ▸). Form A is more thermodynamically stable than the metastable polymorph B; however, Form B is kinetically favored (Német *et al.*, 2009 ▸; Lin *et al.*, 2006 ▸). Metastable polymorph B is the most bioactive and thus used as the famotidine API in commercial anti-GERD medicines, such as Pepcid (Lin, 2014 ▸; Upadhyay *et al.*, 2022 ▸).

Our work began with the successful crystallization and structural analysis of the known famotidine polymorph, Form B. This preliminary work validated our experimental methodology, providing the necessary confidence to tackle the structural elucidation of more com­plex famotidine com­pounds, particularly those relevant to the activated PADs. We significantly expanded the solid-state chemistry of famotidine by determining the crystal structures of three new salts/polymorphs: a famotidine hydro­chloride polymorph, a famotidine hydro­chloride hemihydrate, and a famotidine nitrate salt. Furthermore, we report an unexpected chemical transformation of the API. Under the crystallization conditions em­ployed, we observed the acidic hydrolysis of the amidine N atom, resulting in its replacement by a carbonyl group.

## Experimental

### Chemicals and materials

Famotidine (CAS No. 76824-35-6, >98% pure, HPLC grade) was purchased from TCI America (Portland, OR) and 200 proof ethyl alcohol (CAS No. 64-17-5, >99.98% ACS grade) was purchased from Pharmo-Aaper (Brookfield, CT); both were used as received. Methanol (CAS No 67-56-1, ≥99.8% ACS grade) was purchased from Sigma–Aldrich, Inc. (St Louis, MO) and used as received. Hydro­chloric acid (CAS No. 7647-01-0, Fisher Chemical, 33-38%, technical grade) and nitric acid (CAS No. 7697-37-2, 69–70%, technical grade) were purchased from Fisher Scientific (Hanover Park, IL) and diluted to 3 and 0.1 *M*, respectively. Kimble 20 ml scintillation vials were purchased from Avantar–VWR (Allentown, PA) and Fisherbrand Shell Type 1 glass vials (15 × 45 mm) were purchased from Fisher Scientific (Hanover Park, IL).

### General crystallization procedure

Following the solubility products outlined by Takebayashi and co-workers (Takebayashi *et al.*, 2021 ▸), that famotidine is less soluble in an ethano­lic solution than in a methano­lic solution, we prepared our acidified methano­lic solutions in a 20 ml vial, carefully layered ethanol onto this mixture, and allowed the solution to equilibrate. Upon standing for several days, crystals were found to form and were inspected under a microscope and on the diffractometer to determine what species were present. Unit-cell determinations yielding known parameters were discarded.

#### Famotidine hydro­chloride (I)

Single crystals of famotidine hydro­chloride (**I**) were grown by dissolving famotidine (88 mg, 0.36 mmol) in methanol (3 ml) and acidifying with 3 *M* hydro­chloric acid until a pH of 2 was obtained. Ethanol (7 ml) was layered on the methanol solution in a capped 20 ml vial at room tem­per­a­ture and allowed to stand, yielding the colorless block-like crystals that were analyzed. Colorless needle-like crystals of the known polymorph of famotidine hydrochloride (Ishida *et al.*, 1989 ▸) were also identified and characterized from the bulk sample.

#### Famotidine hydro­chloride hydrate (II) and hydrolyzed famotidine hydro­chloride sesquihydrate (IV)

Famotidine hydro­chloride (18 mg, 0.06 mmol) was dis­solved in 3 ml methanol and was acidified with 3 *M* hydro­chloric acid until a pH of 1 was obtained. Single crystals of **II** and **IV** were both grown by liquid diffusion of ethanol (7 ml) into the acidified methano­lic solution of famotidine hydro­chloride in a sealed 20 ml vial, at room tem­per­a­ture upon standing over one week.

#### Famotidine nitrate (III)

Colorless block-like crystals of famotidine nitrate (**III**) were obtained by liquid diffusion of ethanol (7 ml) into a 3 ml methanol solution of famotidine hydro­chloride (16 mg, 0.05 mmol) in a capped 20 ml vial that was mildly acidified with 0.1 *M* nitric acid at room tem­per­a­ture.

### Refinement

Crystal data, data collection and structure refinement details are summarized in Table 1 ▸. H atoms bonded to C atoms were included in geometrically calculated positions with a riding model [C—H = 0.95 (aromatic) and 0.99 Å (methyl­ene); *U*_iso_(H) = 1.2*U*_eq_(C)]. H atoms bonded to N and O atoms were located from a difference Fourier map. For com­plex **I**, these H atoms were refined freely. A riding model for these H atoms was used for **II**; when freely refined, some of the H-atom positions refined to unreasonable positions. H atoms bonded to N atoms in com­plex **III** were treated with a mixture of freely refined and riding models, depending on how they behaved during refinement. Because of the lower quality of the data for com­plex **IV**, all H atoms were refined with a riding model. The disordered amidinate N atom (N14/N14*A*) in com­plex **II** was modeled over two positions at 50% occupancy. The positions for the two sites were observed in a difference Fourier map. It should be noted that the crystals for com­pound **IV** were particularly challenging. Multiple attempts were made to obtain a suitably diffracting sample that still required the extra X-ray intensity provided by a Diamond micro-focus copper source. Many of these crystallizations were serendipitous. Furthermore, this com­pound suffers from solvent loss during mounting that reduced the data quality. The structural model remains accurate, as atom types were differentiated during refinement, most significantly in the exchange of nitro­gen for oxygen.

## Results and discussion

**Figure 1:**
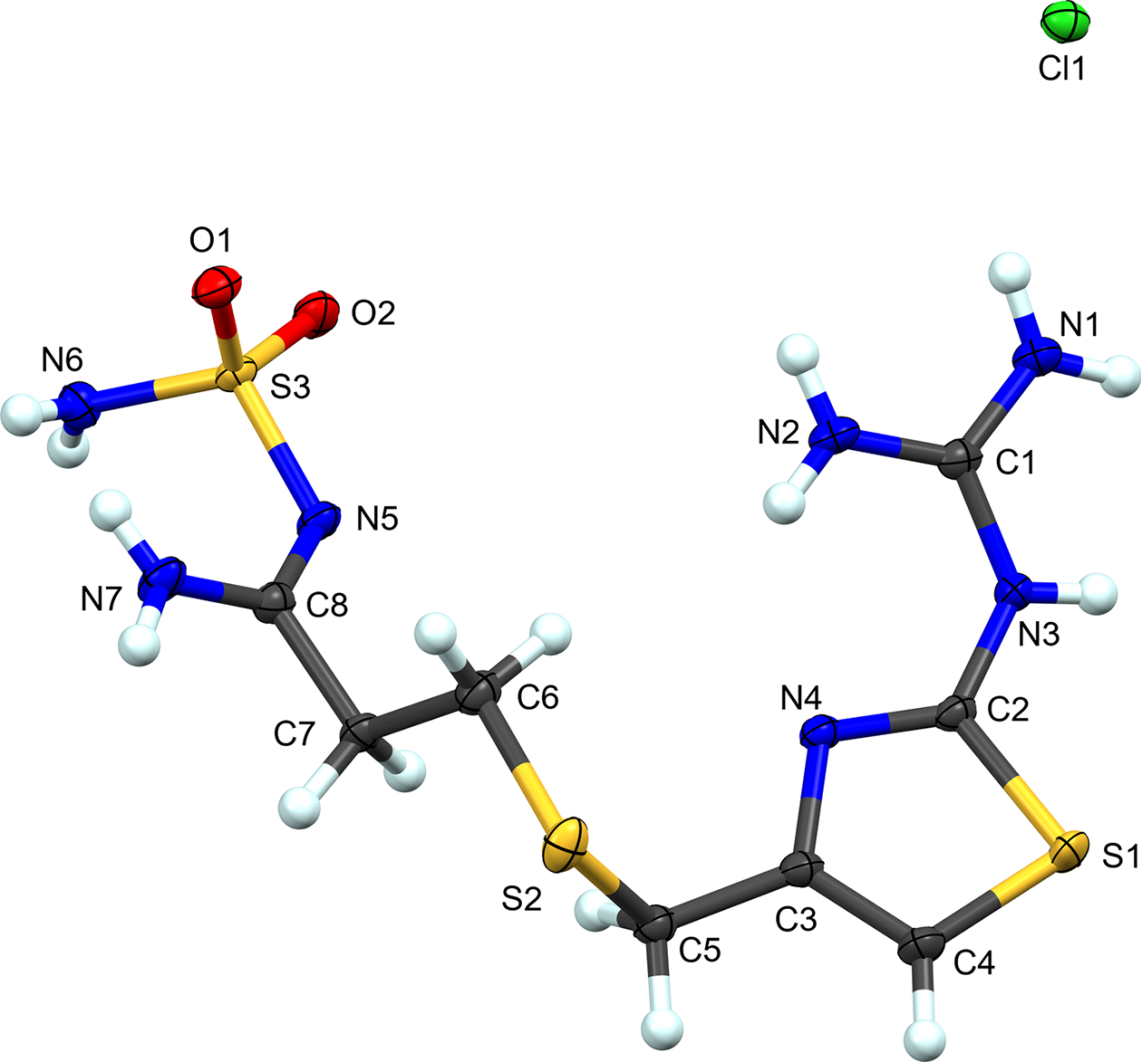
The atom-labeling scheme for **I**. Atomic displacement ellipsoids for non-H atoms are depicted at the 50% probability level and H atoms are shown as spheres of an arbitrary radius.

Famotidine hydro­chloride (**I**) is a new polymorph of the salt (Fig. 1 ▸). The reported structure crystallizes in the *C*-centered monoclinic space group *Cc* (Ishida *et al.*, 1989 ▸), in contrast with the primitive monoclinic *P*2_1_/*n* system reported here (Table 1 ▸). The significant structural difference between the two mol­ecules is the orientation of the sulfamolylpropionamidine moiety. This moiety is rotated ∼112° at the α-carbon (Fig. S1 in the supporting information) with respect to the thia­zole moiety. In both cases, protonation has occurred at guanidine atom N3. In contrast with the two known forms of famotidine, the sulfamolylpropionamidine chain in **I** is extended away from the thia­zole ring. In Form A, the sulfamolylpropionamidine group extends away from the thia­zole then curves back around forming a ‘spoon’-like shape when viewed edge on. In Form B, the chain curves back toward the thia­zole ring forming a ‘C’-shape when viewed edge on (Fig. S2).

**Figure 2:**
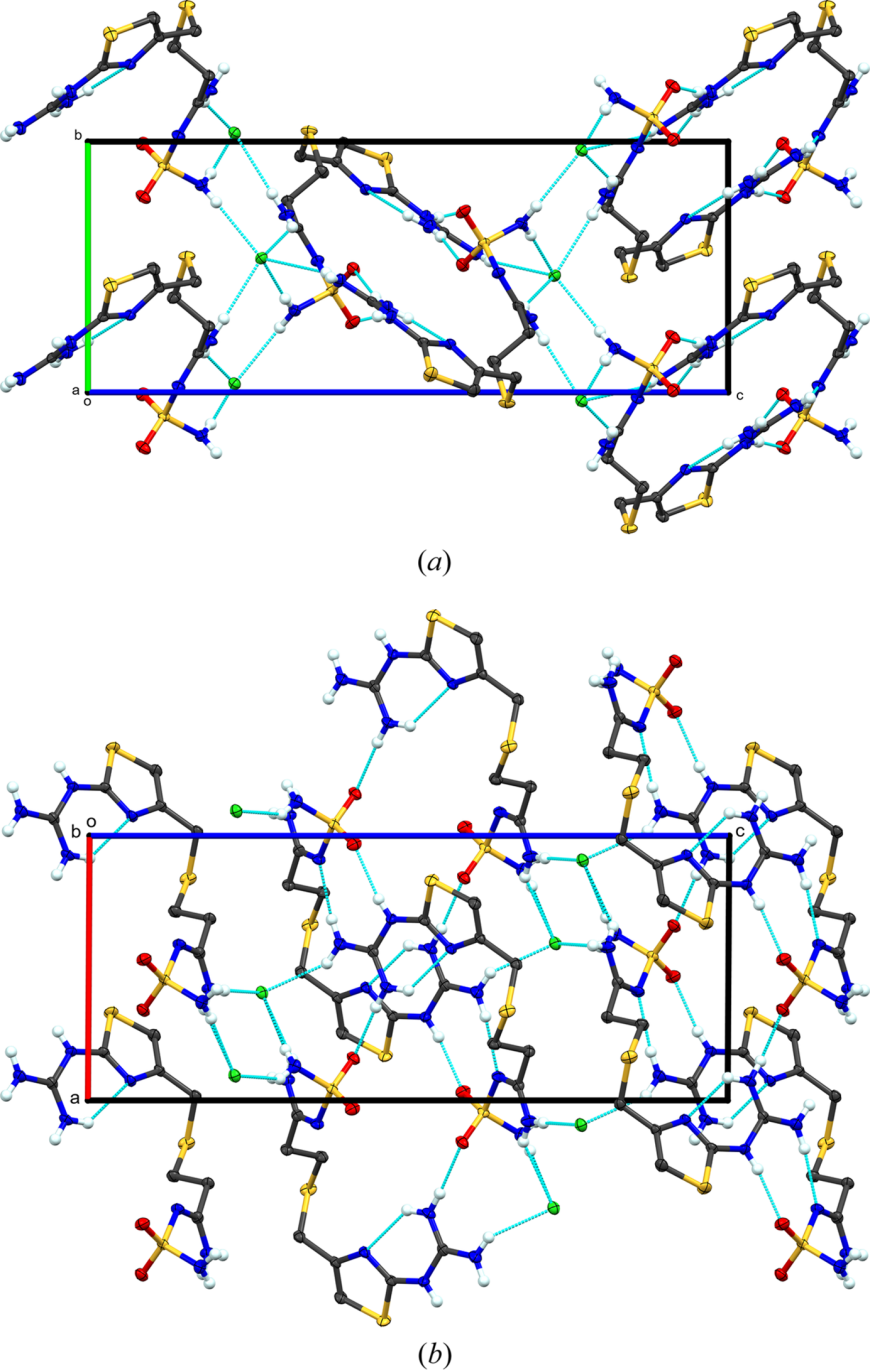
Packing diagram of **I**, viewed along (*a*) the *a* axis and (*b*) the *b* axis. Blue dashed lines represent hy­dro­gen-bond inter­actions. Atomic displacement ellipsoids are shown at the 50% probability level. Only H atoms involved in hy­dro­gen bonding are shown.

Regarding the extended structure of **I**, all donors and acceptors, except the S atoms, are involved in hy­dro­gen bonding (Table 2 ▸ and Fig. 2 ▸). There is one intra­molecular hy­dro­gen bond from guanidine atom N2 to thia­zole atom N4. The guanidine atoms N1 and N3 form a hy­dro­gen bond to the sulfamolyl group of a neighboring cation related by inversion symmetry [N1⋯N5^i^ and N3⋯O2^i^; symmetry code: (i) −*x* + 1, −*y* + 1, −*z* + 1]. The neighboring cation necessarily has reciprocating hy­dro­gen bonds from its guanidine to the standard mol­ecule’s sulfamolyl group. Guanidine atom N2 forms a hy­dro­gen bond to sulfamolyl atom O2^ii^ of a different neighboring cation [symmetry code: (ii) −*x* + 2, −*y* + 1, −*z* + 1]. Atom N1 com­pletes its hy­dro­gen bonding with a contact to the chloride ion (N1⋯Cl1). The chloride ion serves as an acceptor for five hy­dro­gen bonds from four different cations: the noted N1⋯Cl1 contact, inter­actions from N6 of two different symmetry-related cations, and from N7 of two other different famotidine cations. The result of these inter­actions is a hy­dro­gen-bonded chain of chloride ions along the screw axis parallel to the *b* axis. The hy­dro­gen bonds to the neighboring famotidine cations extend this into a three-dimensional network. Graph-set analysis reveals 40 different inter­actions in the solid state, which are beyond utility to discuss here (Etter *et al.*, 1990 ▸).

**Figure 3:**
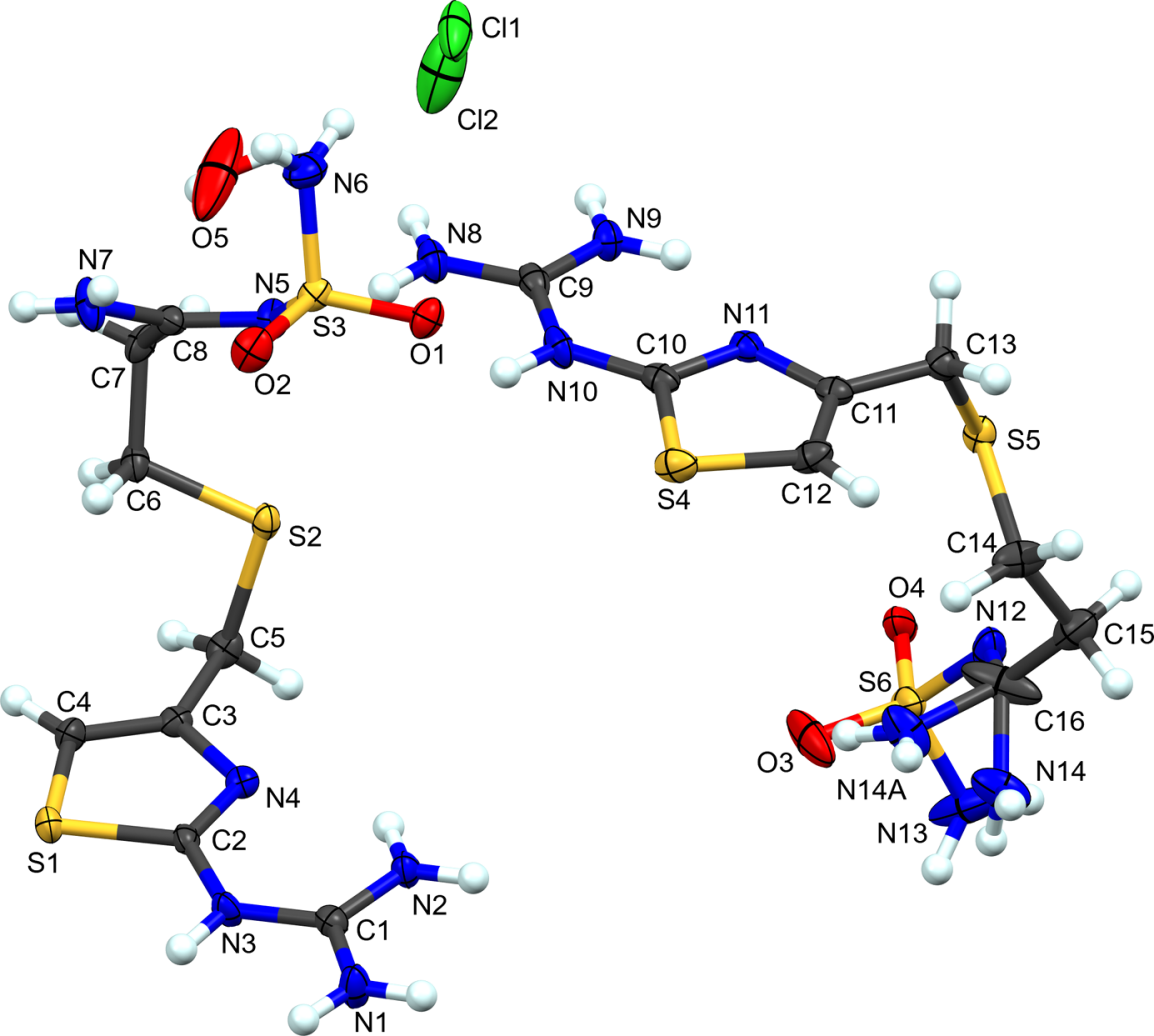
The atom-labeling scheme for **II**. Atomic displacement ellipsoids for non-H atoms are depicted at the 50% probability level and H atoms are shown as spheres of an arbitrary radius.

Complex **II** represents the first structural characterization of a hydro­chloride hydrate of famotidine (Fig. 3 ▸). Formally the structure is a hemihydrate with one water mol­ecule present in the standard unit per two famotidine hydro­chloride salts. One of the two cations has positional disorder at the amidine N atom (N14/N14*A*). In all other respects, the famotidine cations are essentially identical, with only small deviations in the sulfamolylpropionamidine chain when overlaid at the thia­zole group (Fig. S3).

In contrast with the hydro­chloride **I**, there are several intra­molecular hy­dro­gen bonds within the famotidine cations in **II**: the same guanidine-to-thia­zole N-atom hy­dro­gen bond exists (N2⋯N4/N9⋯N11), and a second is a hy­dro­gen bond from amidine atom N7/N14*A* to nearby sulfonamide atom O2/O3, respectively, of the same cation (Table 3 ▸). The disordered amidine atom N14/N14*A* satisfies several different hy­dro­gen-bond inter­actions. The N14 position forms two pairs of bifurcated hy­dro­gen bonds. The first is an intra­molecular hy­dro­gen bond to sulfonamide atom N13 and an inter­molecular contact with Cl1^vi^ [symmetry code: (vi) *x* + 1, *y* + 1, *z*]. However, the former is less likely to be a firm electrostatic inter­action due to directionality. The second H atom forms contacts with the water of crystallization (O5^viii^) and the second chloride (Cl2^viii^) [symmetry code: (viii) *x*, *y* + 1, *z*]. When the N atom is at the N14*A* site, it forms the hy­dro­gen bond previously noted, and an inter­molecular hy­dro­gen bond to O2^i^ of a sulfonamide group on a neighboring cation [symmetry code: (i) −*x* + 1, −*y* + 1, −*z* + 1].

**Figure 4:**
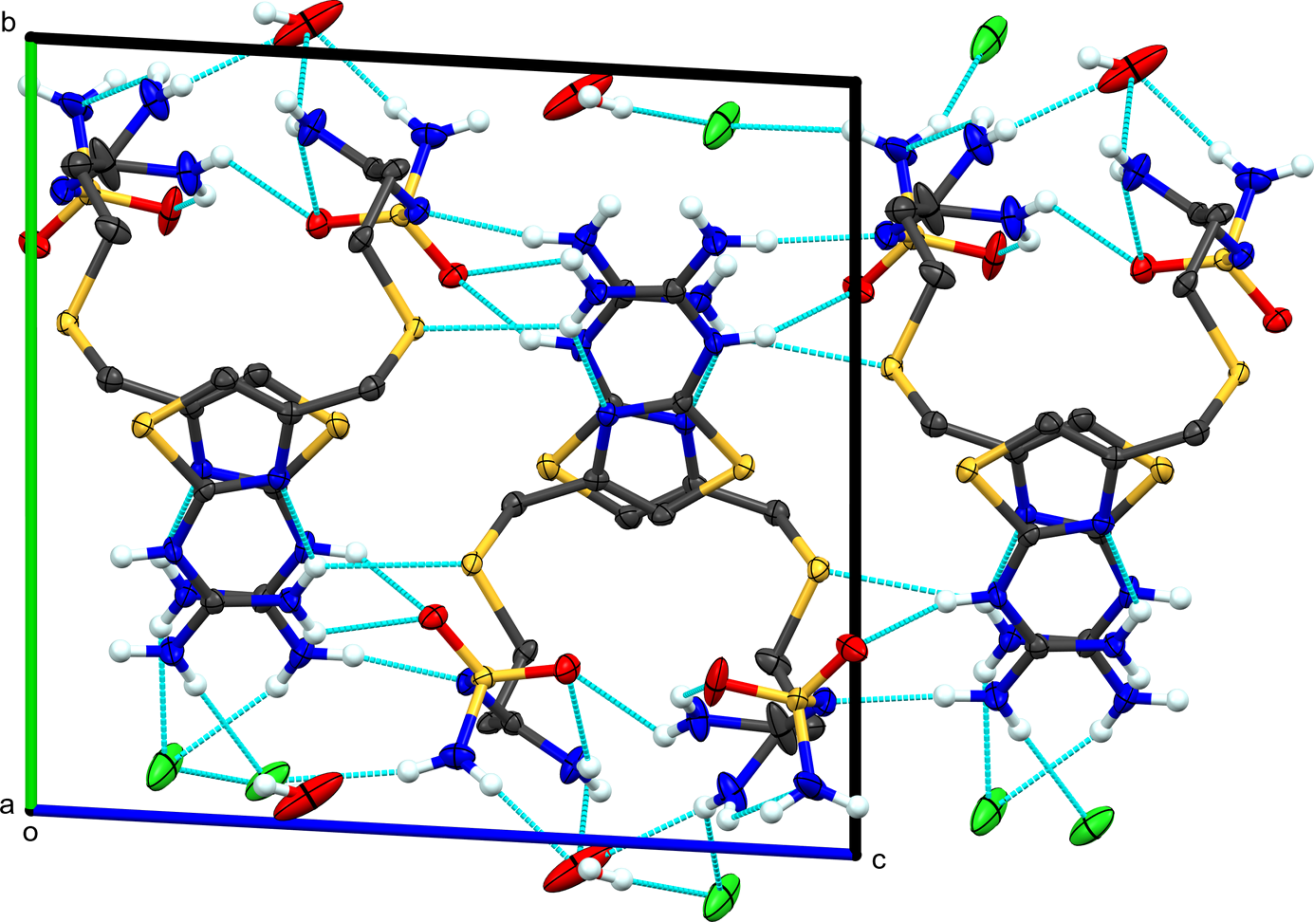
Packing diagram of **II**, viewed along the *a* axis. Blue dashed lines represent hy­dro­gen-bond inter­actions. Atomic displacement ellipsoids are shown at the 50% probability level. Only H atoms involved in hy­dro­gen bonding are shown.

The presence of two symmetry-independent famotidine hydro­chloride com­plexes and a water of crystallization create a plethora of hy­dro­gen-bond inter­actions (Table 3 ▸ and Fig. 4 ▸). This discussion will focus on the significant differences across this series of materials. Examining the chloride ions and water mol­ecule, Cl1 serves as an acceptor for five hy­dro­gen bonds and Cl2 accepts six hy­dro­gen bonds. The water of crystallization is a donor in two hy­dro­gen bonds to each chloride and is an acceptor of three hy­dro­gen bonds from the two sulfamolyl N atoms of one cation and the sulfonamide N atom of a second cation (N6, N7, and N14, successively). The guanidine intra­molecular hy­dro­gen bond (above) is bifurcated with a contact to an S atom on an adjacent cation [N2⋯S2^i^ and N9⋯S5^ii^; symmetry code: (i) −*x* + 1, −*y* + 1, −*z* + 1; (ii) *x*, *y*, *z* + 1]. This is in contrast with **I**, in which the S atoms are not part of the hy­dro­gen-bonding network. These N—H⋯S hy­dro­gen bonds are self-com­plementary related across inversion centers. The sulfonamide N atoms (N6 and N13) differ, in their inter­molecular contacts. Atom N6 forms hy­dro­gen bonds with water O5^iv^ and Cl1, whereas N13 forms hy­dro­gen bonds to both chloride ions, like that of the sulfamido N atom in **I** [symmetry code: (iv) −*x* + 1, −*y*, −*z* + 1].

**Figure 5:**
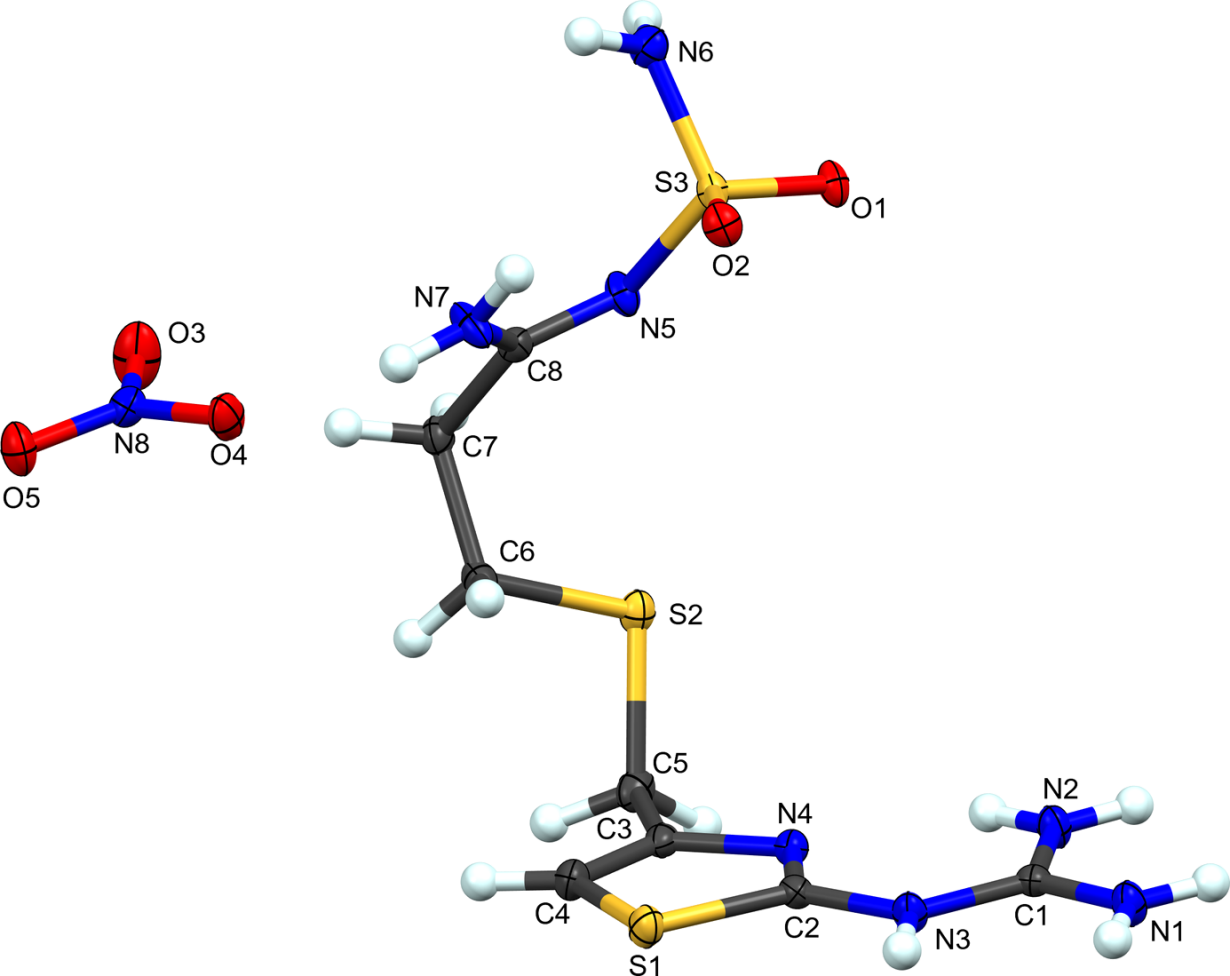
The atom-labeling scheme for **III**. Atomic displacement ellipsoids for non-H atoms are depicted at the 50% probability level and H atoms are shown as spheres of an arbitrary radius.

Complex **III** is formally the hydro­nitrate salt of famotidine (Fig. 5 ▸). As with the hydro­chloride salts, protonation occurs at atom N3 of the guanidine moiety. With regard to the crystal morphology, this nitrate forms large block-like crystals com­pared with the rod-like crystals observed for the other com­plexes presented here. This implies potential utility in separations with this different larger morphology. Nitrates are also not inherently haza­rdous to biological systems and may present an alternative for API development. Like famotidine Form B, the sulfonamide group is curved back towards the thia­zole ring forming a slightly open ‘C’ shape in the solid state.

**Figure 6:**
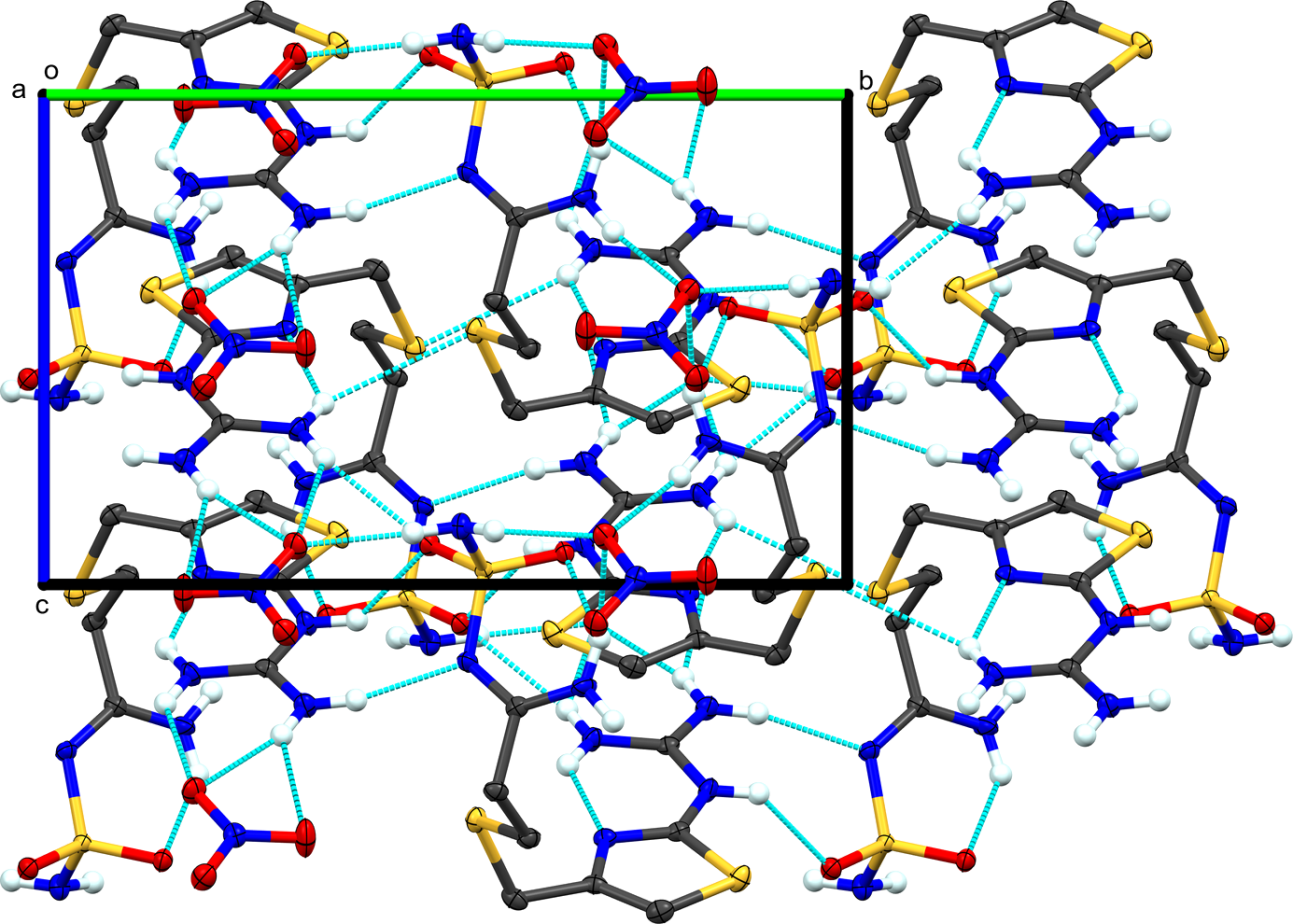
Packing diagram of **III**, viewed along the *a* axis. Blue dashed lines represent hy­dro­gen-bond inter­actions. Atomic displacement ellipsoids are shown at the 50% probability level. Only H atoms involved in hy­dro­gen bonding are shown.

Germane to these com­plexes, there is an intra­molecular hy­dro­gen bond from guanidine atom N2 to thia­zole atom N4 (Table 4 ▸). Like **II**, atom N2 also forms a bifurcated hy­dro­gen bond to S2^iv^ on a neighboring cation [symmetry code: (iv) −*x* + 1, −*y* + 1, −*z* + 1]. Another similarity with **II** is the intra­molecular hy­dro­gen bond from N7 to O2, in contrast with **I**. Predictably, the nitrate anion serves as a hy­dro­gen-bond hub in this structure. Atom O3 is an acceptor of one hy­dro­gen bond that is shared (bifurcated) with O5. Atoms O4 and O5 both accept three hy­dro­gen bonds. Atom O4 is an acceptor for hy­dro­gen bonds from sulfamolyl atoms N6 and N7 of one cation, and N7 of a second famotidine cation. The bifurcated hy­dro­gen bond between O3 and O5 originates from guanidine atom N1 on a neighboring cation. Both H atoms on guanidine atom N2 form bifurcated hy­dro­gen bonds. One is the intra­molecular hy­dro­gen bond described above, that is shared with S2^iv^. The second H atom forms contacts with nitrate atom O5^i^ and sulfonamide atom O1^iii^ of a second neighboring cation, that also accepts a second hy­dro­gen bond from N3 from a different cation [symmetry codes: (i) *x* + 1, −*y* + 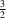, *z* − 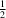; (iii) −*x* + 1, −*y* + 1, −*z*]. The third hy­dro­gen bond to nitrate atom O5 is from sulfonamide atom N6. These various inter­actions are highlighted in Fig. 6 ▸.

**Figure 7:**
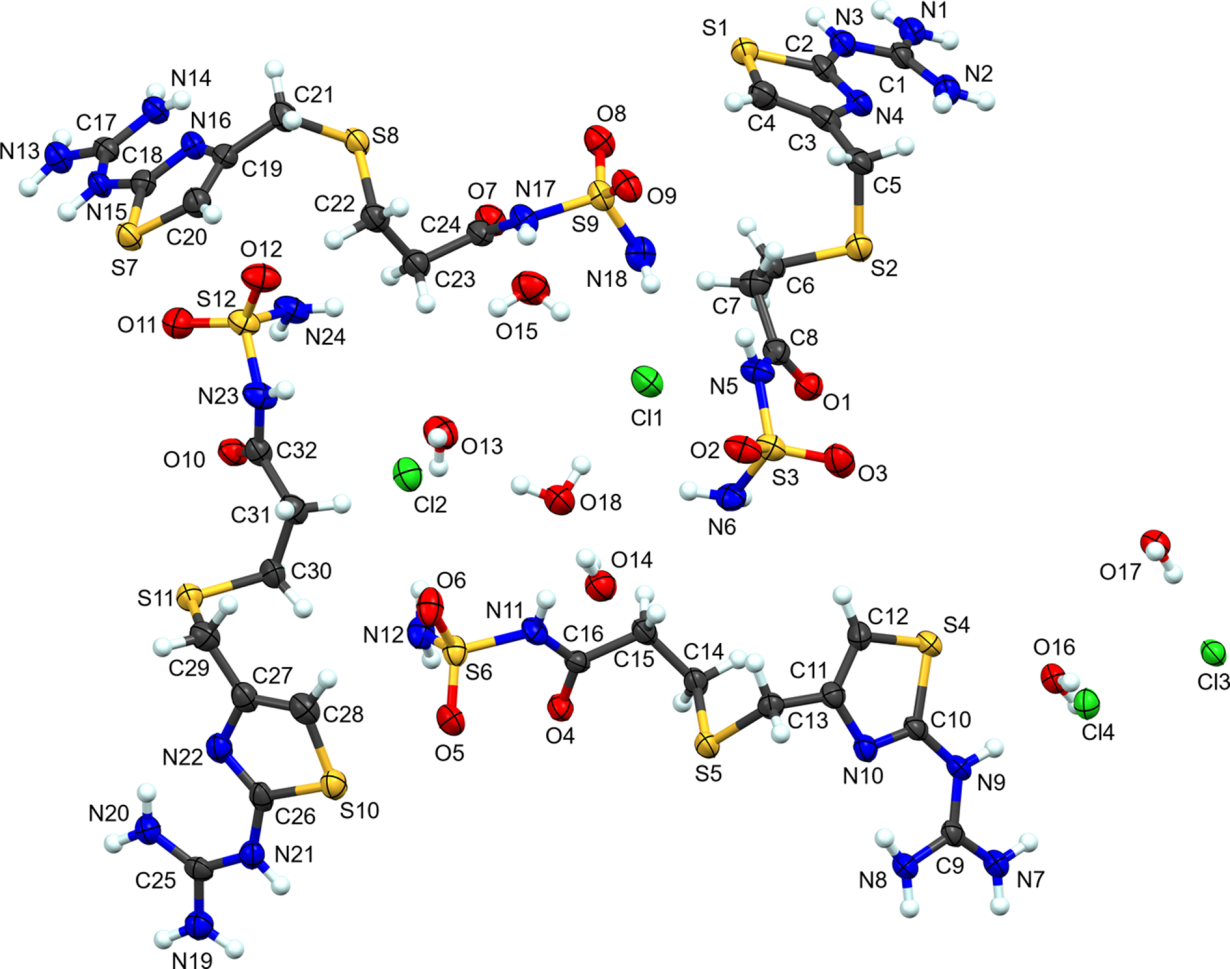
The atom-labeling scheme for **IV**. Atomic displacement ellipsoids for non-H atoms are depicted at the 50% probability level and H atoms are shown as spheres of an arbitrary radius.

Complex **IV** presents an unusual modification of the parent famotidine com­pound. The amidine N atom (N7 in the parent com­pound) has been replaced with an O atom and sulfonamide atom N6 has been protonated (Fig. 7 ▸). Evidence for this modification appears in the form of the hy­dro­gen bonds in which these atoms are involved (see below for details). Given that hydro­chloric acid is present in the crystallization medium, presumably this is an acid hydrolysis. Furthermore, this is an example of a high-*Z*′ structure with four crystallographically independent cations and associated anions in the asymmetric unit. It is also a sesquihydrate, with six unique water mol­ecules in the standard unit (1.5 water mol­ecules per salt). Inspection of the differences between the four cations is highlighted in the overlay (Fig. S4). Mol­ecules 2 (S4) and 4 (S10) are remarkably similar. In contrast, mol­ecules 1 (S1) and 3 (S7) have a similar orientation along the propionamide chain and deviate at C6/C22. The torsion angles along the propionamide C—S—C—C chain and N(thia­zole)—C(thia­zole)—C—S highlight these differences (Table 5 ▸).

Complex **IV** appears to be structurally similar to Famotidine Related Compound C (or Famotidine Impurity C), a known degradation product of the API famotidine (USP-NF, 2020 ▸). The com­pound’s chemical name is 3-[({2-[(di­amino­methyl­idene)amino]-1,3-thia­zol-4-yl}meth­yl)sulfan­yl]-*N*-sulfamoylpropanamide (C_8_H_14_N_6_O_3_S_3_). Famotidine Impurity C is pri­marily formed through the hydrolysis of famotidine (Junnarkar & Stavchansky, 1995 ▸; Suleiman *et al.*, 1989 ▸), which distinguishes it from a synthetic impurity. Therefore, it is reasonable to conclude that **IV** is a hydrated salt of the freebase Famotidine Impurity C, a known and previously characterized degradation product of the API famotidine.

**Figure 8:**
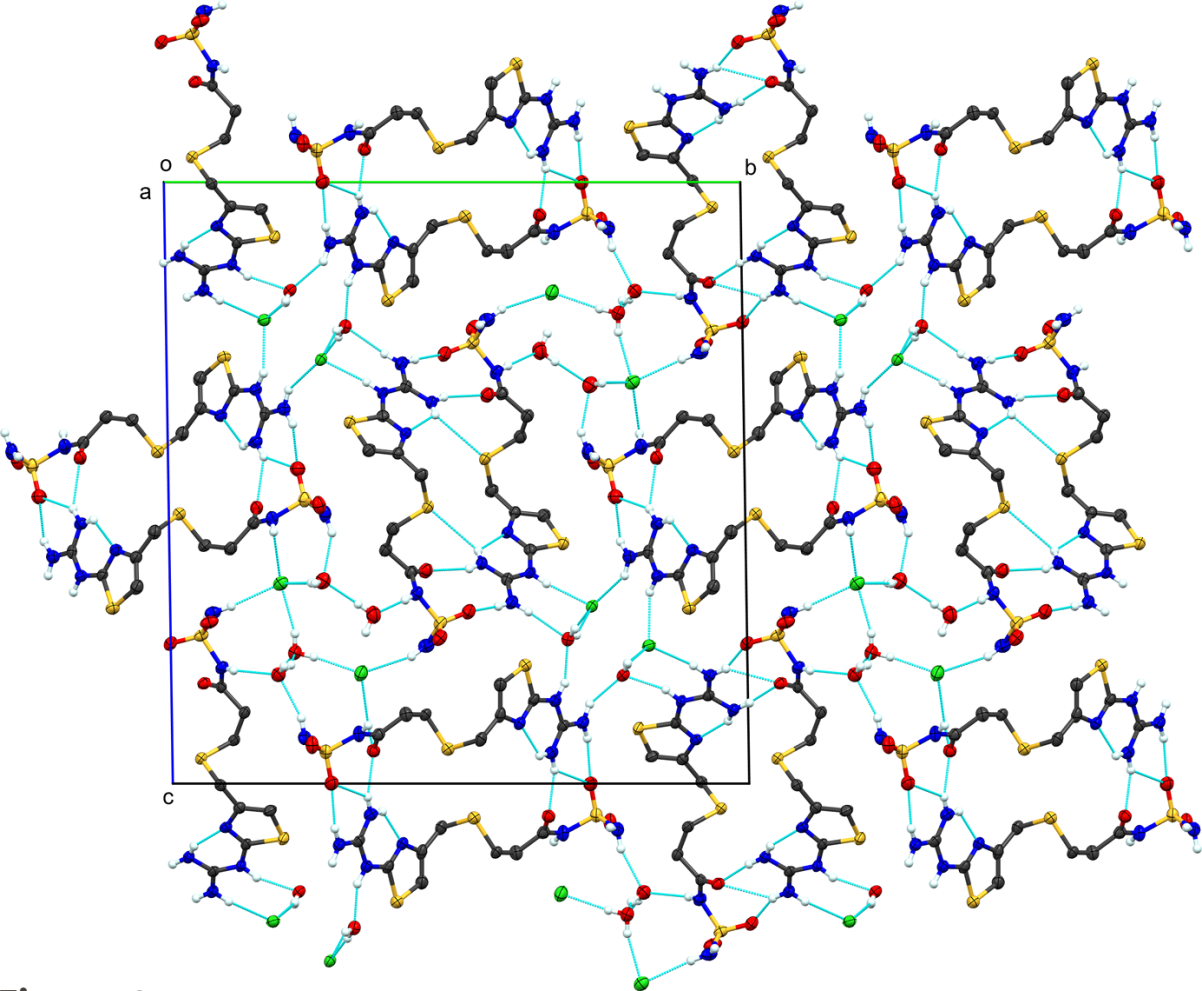
Packing diagram of **IV**, viewed along the *a* axis. Blue dashed lines represent hy­dro­gen-bond inter­actions. Atomic displacement ellipsoids are shown at the 50% probability level. Only H atoms involved in hy­dro­gen bonding are shown.

With four crystallographically-independent cations, associated anions, and solvent mol­ecules, the extended structure of **IV** has numerous inter­molecular inter­actions (Table 6 ▸ and Fig. 8 ▸). Thus, discussion will be restricted to the more salient features of the packing. From one perspective, each of the cations forms a centrosymmetric self-dimer. The dimers are stacked along the *a* axis. Ignoring the anion and waters of crystallization, these four stacks of mol­ecules form a her­ring­bone pattern. Located within channels formed between the herringbone array are two channels. These channels are populated with hy­dro­gen-bonded chains of water mol­ecules and Cl atoms. One chain consists of chloride ions Cl1 and Cl2, along with water mol­ecules O13, O14, O15, and O18. The second channel contains the two remaining chloride ions (Cl3 and Cl4) and water mol­ecules O16 and O17. The ubiquitous guanidine-to-thia­zole N-atom hy­dro­gen bond is present in all four cations. The hy­dro­gen-bonded self-dimers, for three of the four modified famotidine mol­ecules, are formed by hy­dro­gen bonds from the guanidine moiety on one mol­ecule to a sulfonamide O atom and the adjacent carbonyl O atom that has replaced the amidinate N atom by hydrolysis. The outlier is the chain formed by the fourth mol­ecule (S7) that forms hy­dro­gen-bond contacts with two different centrosymmetric cations. The guanidine moiety forms hy­dro­gen bonds to the sulfonamide and adjacent carbonyl O atom on one cation (N13⋯O8^vi^ and N14⋯O7^vi^). Unlike the other three cations, one guanidine N atom (N14) forms a hy­dro­gen bond to S8^viii^ of a second cation [symmetry codes: (vi) −*x* + 2, −*y* + 1, −*z* + 1; (viii) −*x* + 1, −*y* + 1, −*z* + 1]. This results in the sulfonamide and carbonyl O atoms at the terminus of the standard cation accepting hy­dro­gen bonds from the guanidine of this second hy­dro­gen-bonded inversion-related cation. Despite the lack of translation symmetry that is typical for such formations, this chain of hy­dro­gen-bonded mol­ecules adopts a helical motif.

## Conclusion

Famotidine, a widely distributed anti-GERD drug typically formulated as the hydro­chloride salt, was systematically investigated across a range of crystallization conditions and acidities. As a result of this study, we successfully characterized the crystal structures of four new com­plexes: a polymorph of the famotidine hydro­chloride salt, a hemihydrate of the famotidine hydro­chloride salt, and a famotidine hydro­nitrate salt. The fourth structure represents a hydrolyzed salt of a known famotidine degradation product; it is formed through the replacement of the amidine N atom by a carbonyl group, accom­panied by protonation at a neighboring N atom. All characterized structures are stabilized *via* extensive intra- and inter­molecular hy­dro­gen-bonded networks. These structural elucidations provide the necessary foundation for characterizing more com­plex systems, including the colored famotidine–metal com­plexes found on the activated anti-GERD PADs.

## Supplementary Material

Crystal structure: contains datablock(s) I, II, III, IV, global. DOI: 10.1107/S2053229626004122/wv3024sup1.cif

Structure factors: contains datablock(s) I. DOI: 10.1107/S2053229626004122/wv3024Isup2.hkl

Structure factors: contains datablock(s) II. DOI: 10.1107/S2053229626004122/wv3024IIsup3.hkl

Structure factors: contains datablock(s) III. DOI: 10.1107/S2053229626004122/wv3024IIIsup4.hkl

Structure factors: contains datablock(s) IV. DOI: 10.1107/S2053229626004122/wv3024IVsup5.hkl

Supporting information file. DOI: 10.1107/S2053229626004122/wv3024Isup6.cml

Supporting information file. DOI: 10.1107/S2053229626004122/wv3024IIsup7.cml

Supporting information file. DOI: 10.1107/S2053229626004122/wv3024IIIsup8.cml

Supporting information file. DOI: 10.1107/S2053229626004122/wv3024IVsup9.cml

Additional figures. DOI: 10.1107/S2053229626004122/wv3024sup10.pdf

CCDC references: 2547671, 2547672, 2547673, 2547674

## Figures and Tables

**Table 1:** Experimental details Experiments were carried out at 120 K. Absorption was corrected for by numerical methods (*SADABS*; Krause *et al.*, 2015 ▸).

	**I**	**II**	**III**	**IV**
Crystal data				
Chemical formula	C_8_H_16_N_7_O_2_S_3_^+^·Cl^−^	C_8_H_16_N_7_O_2_S_3_^+^·Cl^−^·0.5H_2_O	C_8_H_16_N_7_O_2_S_3_^+^·NO_3_^−^	C_8_H_15_N_6_O_3_S_3_^+^·Cl^−^·1.5H_2_O
*M*_r_	373.91	382.91	400.47	401.91
Crystal system, space group	Monoclinic, *P*2_1_/*n*	Triclinic, *P* 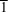	Monoclinic, *P*2_1_/*c*	Triclinic, *P* 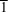
*a*, *b*, *c* (Å)	8.8712 (14), 8.4069 (13), 21.423 (3)	8.5104 (3), 13.8816 (4), 14.1753 (5)	13.5616 (16), 13.9590 (17), 8.504 (1)	5.1449 (6), 25.285 (2), 26.306 (2)
α, β, γ (°)	90, 90.357 (3), 90	92.178 (1), 92.655 (1), 107.297 (1)	90, 90.597 (2), 90	89.017 (7), 87.760 (8), 86.803 (8)
*V* (Å^3^)	1597.7 (4)	1594.84 (9)	1609.8 (3)	3413.9 (6)
*Z*	4	4	4	8
Radiation type	Mo *K*α	Mo *K*α	Mo *K*α	Cu *K*α
μ (mm^−1^)	0.65	0.65	0.50	5.69
Crystal size (mm)	0.20 × 0.11 × 0.06	0.18 × 0.12 × 0.07	0.21 × 0.16 × 0.08	0.20 × 0.06 × 0.02

Data collection				
Diffractometer	Bruker D8	Bruker D8	Bruker D8	Bruker Venture
*T*_min_, *T*_max_	0.945, 0.989	0.830, 0.933	0.927, 0.989	0.475, 0.757
No. of measured, independent and observed [*I* > 2σ(*I*)] reflections	23845, 3963, 3237	40692, 7938, 6530	24503, 4017, 3502	82198, 12581, 8070
*R*_int_	0.040	0.044	0.029	0.219
(sin θ/λ)_max_ (Å^−1^)	0.667	0.667	0.668	0.608

Refinement				
*R*[*F*^2^ > 2σ(*F*^2^)], *wR*(*F*^2^), *S*	0.028, 0.069, 1.04	0.062, 0.130, 1.09	0.027, 0.068, 1.04	0.074, 0.190, 1.04
No. of reflections	3963	7938	4017	12581
No. of parameters	224	398	223	876
H-atom treatment	H atoms treated by a mixture of independent and constrained refinement	H-atom parameters constrained	H atoms treated by a mixture of independent and constrained refinement	H atoms treated by a mixture of independent and constrained refinement
Δρ_max_, Δρ_min_ (e Å^−3^)	0.33, −0.37	1.37, −1.79	0.35, −0.37	0.48, −0.52

Computer programs: *APEX4* (Bruker, 2021 ▸), *SAINT* (Bruker, 2021 ▸), *SHELXT2018* (Sheldrick, 2015*a* ▸), *SHELXL2019* (Sheldrick, 2015*b* ▸), *Mercury* (Macrae *et al.*, 2020 ▸), and *FinalCIF* (Kratzert, 2025 ▸).

**Table 2:** Hydrogen-bond geometry (Å, °) for **I**[Chem scheme1]

*D*—H⋯*A*	*D*—H	H⋯*A*	*D*⋯*A*	*D*—H⋯*A*
N1—H1*A*⋯Cl1	0.84 (2)	2.44 (2)	3.1828 (15)	149 (2)
N1—H1*B*⋯N5^i^	0.87 (2)	2.14 (2)	2.999 (2)	169 (2)
N2—H2*A*⋯O1^ii^	0.83 (2)	2.09 (2)	2.9253 (18)	174 (2)
N2—H2*B*⋯N4	0.82 (2)	2.18 (2)	2.801 (2)	131.9 (19)
N3—H3⋯O2^i^	0.802 (19)	2.02 (2)	2.8115 (19)	168.2 (19)
N6—H6*A*⋯Cl1^iii^	0.87 (2)	2.42 (2)	3.1863 (16)	148.6 (19)
N6—H6*B*⋯Cl1^ii^	0.76 (2)	2.47 (2)	3.2230 (18)	174 (2)
N7—H7*A*⋯Cl1^ii^	0.82 (2)	2.66 (2)	3.3584 (16)	144.1 (17)
N7—H7*A*⋯O1	0.82 (2)	2.48 (2)	2.9627 (19)	118.4 (16)
N7—H7*B*⋯Cl1^iv^	0.86 (2)	2.40 (2)	3.2644 (16)	176.7 (18)

Symmetry codes: (i) 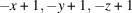; (ii) 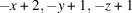; (iii) 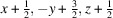; (iv) 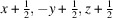.

**Table 3:** Hydrogen-bond geometry (Å, °) for **II**[Chem scheme1]

*D*—H⋯*A*	*D*—H	H⋯*A*	*D*⋯*A*	*D*—H⋯*A*
N1—H1*A*⋯Cl1^i^	0.88	2.31	3.177 (3)	170
N1—H1*B*⋯N12^ii^	0.88	2.08	2.959 (4)	176
N2—H2*A*⋯O1^i^	0.88	2.57	3.233 (4)	132
N2—H2*B*⋯S2^iii^	0.88	2.92	3.591 (3)	134
N2—H2*B*⋯N4	0.88	2.07	2.728 (4)	131
N3—H3⋯O4^ii^	0.88	2.01	2.801 (4)	150
N6—H6*A*⋯O5^iv^	0.88	2.12	2.826 (5)	137
N6—H6*B*⋯Cl1	0.88	2.44	3.303 (4)	167
N7—H7*A*⋯O2	0.88	2.30	2.882 (4)	123
N7—H7*A*⋯O5^iv^	0.88	2.63	3.315 (5)	136
N7—H7*B*⋯Cl1^iv^	0.88	2.58	3.411 (3)	157
N8—H8*A*⋯Cl2	0.88	2.42	3.226 (3)	152
N8—H8*B*⋯N5	0.88	2.10	2.959 (4)	166
N9—H9*A*⋯Cl2	0.88	2.64	3.388 (3)	144
N9—H9*B*⋯S5^v^	0.88	2.97	3.634 (3)	134
N9—H9*B*⋯N11	0.88	2.07	2.727 (4)	131
N10—H10⋯O1	0.88	2.15	2.821 (4)	133
N13—H13*C*⋯Cl2^vi^	0.88	2.71	3.466 (5)	145
N13—H13*D*⋯Cl2^vii^	0.88	2.48	3.250 (4)	147
N14—H14*E*⋯Cl1^vi^	0.88	2.46	3.131 (6)	134
N14—H14*F*⋯Cl2^viii^	0.88	2.81	3.531 (7)	140
N14—H14*F*⋯O5^viii^	0.88	2.46	3.167 (8)	138
N14*A*—H14*C*⋯O3	0.88	1.82	2.539 (7)	138
N14*A*—H14*D*⋯O2^i^	0.88	2.35	2.999 (7)	131
O5—H5*C*⋯Cl2	0.87	2.27	3.126 (5)	167
O5—H5*D*⋯Cl1^ix^	0.87	2.30	3.168 (4)	175

Symmetry codes: (i) 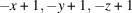; (ii) 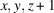; (iii) 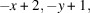

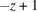
; (iv) 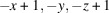; (v) 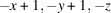; (vi) 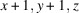; (vii) 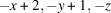; (viii) 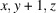; (ix) 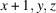.

**Table 4:** Hydrogen-bond geometry (Å, °) for **III**[Chem scheme1]

*D*—H⋯*A*	*D*—H	H⋯*A*	*D*⋯*A*	*D*—H⋯*A*
N1—H1*A*⋯O3^i^	0.88	2.40	3.1392 (16)	141
N1—H1*A*⋯O5^i^	0.88	2.08	2.9302 (16)	161
N1—H1*A*⋯N8^i^	0.88	2.59	3.4547 (17)	170
N1—H1*B*⋯N5^ii^	0.88	2.11	2.9647 (17)	164
N2—H2*A*⋯O1^iii^	0.88	2.37	3.0669 (15)	137
N2—H2*A*⋯O5^i^	0.88	2.62	3.3299 (15)	138
N2—H2*B*⋯S2^iv^	0.88	2.95	3.5664 (13)	128
N2—H2*B*⋯N4	0.88	2.05	2.7199 (16)	132
N3—H3⋯O1^ii^	0.88	2.10	2.7914 (15)	135
N7—H7*A*⋯O2	0.88	2.18	2.8042 (15)	127
N7—H7*A*⋯O4^v^	0.88	2.39	3.0515 (16)	133
N7—H7*B*⋯O4	0.88	2.03	2.8997 (16)	168
N6—H6*A*⋯O5^vi^	0.861 (18)	2.085 (18)	2.8992 (17)	157.6 (16)
N6—H6*B*⋯O4^v^	0.856 (18)	2.115 (19)	2.9184 (16)	156.1 (16)

Symmetry codes: (i) 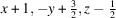; (ii) 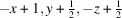; (iii) 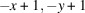, 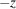; (iv) 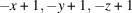; (v) 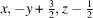; (vi) 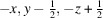.

**Table 5:** Selected torsion angles (°) for **IV**[Chem scheme1]

N4—C3—C5—S2	−77.7 (6)	N16—C19—C21—S8	−75.3 (6)
C5—S2—C6—C7	−85.3 (5)	C21—S8—C22—C23	157.5 (5)
N10—C11—C13—S5	−59.9 (6)	C26—S10—C28—C27	−0.9 (5)
C13—S5—C14—C15	−88.8 (5)	N22—C27—C29—S11	−60.7 (7)

**Table 6:** Hydrogen-bond geometry (Å, °) for **IV**[Chem scheme1]

*D*—H⋯*A*	*D*—H	H⋯*A*	*D*⋯*A*	*D*—H⋯*A*
N1—H1*A*⋯O16^i^	0.87	2.02	2.821 (7)	152
N1—H1*B*⋯O3^ii^	0.87	2.08	2.913 (8)	160
N2—H2*A*⋯N4	0.87	2.05	2.678 (8)	128
N2—H2*B*⋯O1^ii^	0.87	2.06	2.871 (7)	155
N2—H2*B*⋯O3^ii^	0.87	2.61	3.240 (8)	130
N3—H3⋯O17^ii^	0.87	1.96	2.781 (7)	156
N5—H5⋯Cl1^iii^	0.87	2.61	3.262 (6)	133
N6—H6*A*⋯O2^iv^	0.87	1.98	2.810 (7)	159
N6—H6*B*⋯O14^iii^	0.87	2.17	3.007 (8)	162
N7—H7*C*⋯O4^v^	0.87	2.48	3.138 (6)	133
N7—H7*C*⋯O5^v^	0.87	2.18	2.966 (7)	150
N7—H7*D*⋯Cl4^iv^	0.87	2.39	3.245 (5)	168
N8—H8*A*⋯O4^v^	0.87	2.05	2.904 (7)	167
N8—H8*B*⋯N10	0.87	1.93	2.709 (8)	149
N9—H9⋯O16	0.87	1.98	2.817 (6)	161
N11—H11⋯O14^iii^	0.87	2.10	2.862 (7)	146
N12—H12*A*⋯O6^iv^	0.87	1.97	2.798 (7)	158
N12—H12*B*⋯Cl2	0.87	2.41	3.278 (6)	174
N13—H13*C*⋯O8^vi^	0.87	1.94	2.811 (7)	175
N13—H13*D*⋯O17^vii^	0.87	2.14	3.011 (7)	175
N14—H14*C*⋯S8^viii^	0.87	3.00	3.680 (5)	136
N14—H14*C*⋯N16	0.87	2.10	2.750 (7)	131
N14—H14*D*⋯O7^vi^	0.87	2.13	2.932 (7)	154
N15—H15⋯Cl3^ix^	0.87	2.25	3.083 (5)	159
N17—H17⋯O15	0.87	1.90	2.756 (8)	168
N18—H18*A*⋯O9^iv^	0.87	2.05	2.890 (7)	162
N18—H18*B*⋯Cl1	0.87	2.42	3.257 (6)	162
N19—H19*A*⋯Cl3^v^	0.87	2.48	3.213 (6)	142
N19—H19*B*⋯O11^x^	0.87	2.06	2.876 (8)	155
N20—H20*A*⋯N22	0.87	2.07	2.700 (8)	129
N20—H20*B*⋯O10^x^	0.87	2.26	2.964 (7)	137
N20—H20*B*⋯O11^x^	0.87	2.42	3.167 (8)	144
N21—H21⋯Cl4^xi^	0.87	2.34	3.110 (5)	148
N23—H23⋯Cl2^iii^	0.87	2.49	3.294 (6)	154
N24—H24*A*⋯O12^iv^	0.87	1.99	2.807 (8)	157
N24—H24*B*⋯O13^iii^	0.87	2.15	2.933 (8)	150
O13—H13*E*⋯Cl2^iv^	0.85	2.29	3.142 (6)	175
O13—H13*F*⋯Cl2	0.85	2.37	3.200 (6)	164
O14—H14*E*⋯O18	0.85	1.95	2.798 (8)	172
O14—H14*F*⋯O18^iv^	0.85	2.20	2.884 (8)	138
O15—H15*C*⋯Cl1	0.85	2.85	3.335 (6)	118
O15—H15*D*⋯O13^iii^	0.85	1.97	2.813 (8)	175
O16—H16*A*⋯Cl4^iv^	0.85	2.18	2.992 (5)	160
O16—H16*B*⋯Cl4	0.85	2.37	3.195 (5)	164
O17—H17*A*⋯Cl3^iii^	0.85	2.30	3.100 (5)	158
O17—H17*B*⋯Cl3	0.85	2.32	3.165 (5)	173
O18—H18*C*⋯Cl1	0.85	2.20	3.045 (5)	174
O18—H18*D*⋯Cl2	0.85	2.32	3.148 (5)	165

Symmetry codes: (i) 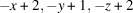; (ii) 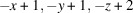; (iii) 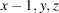; (iv) 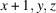; (v) 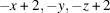; (vi) 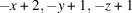; (vii) 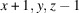; (viii) 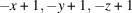; (ix) 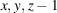; (x) 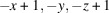; (xi) 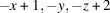.
