# Supplementary material for: Hydro­chlorides, hydrates, hydro­nitrate, and an unanti­cipated hydrolysis product of famotidine
Source: Acta Crystallogr C Struct Chem. 2026 Apr 24;82(Pt 5):241–9. doi: 10.1107/S2053229626004122 (PMC13139895; doi:10.1107/S2053229626004122)
Supplement: Supplementary file 10 [file c-82-00241-sup10.pdf]

Acta Cryst C

STRUCTURAL CHEMISTRY

Volume 82 (2026)

Supporting information for article:

Hydrochlorides, hydrates, hydronitrate, and an unanticipated hydrolysis product of famotidine

MacKenzie C. Weaver, Allen G. Oliver and Toni L. O. Barstis

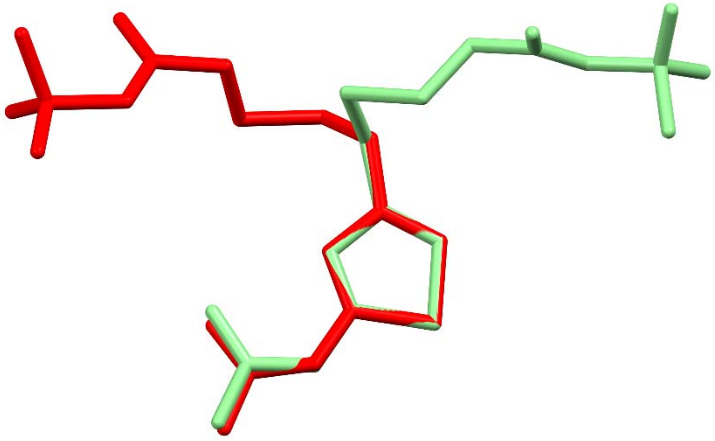

Figure S1 Overlay of the famotidine moieties (**I**, red) and the reported structure (light green; Ishida *et al.*, 1989).

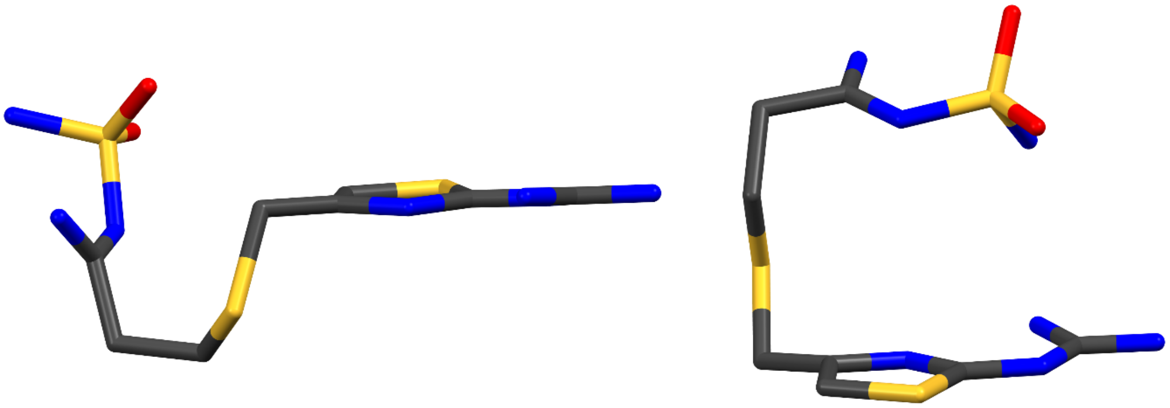

Figure S2 Representations of the two reported forms of famotidine (Left: Form A; Golic *et al.*, 1989; Right: Form B; Shankland *et al.*, 2002).

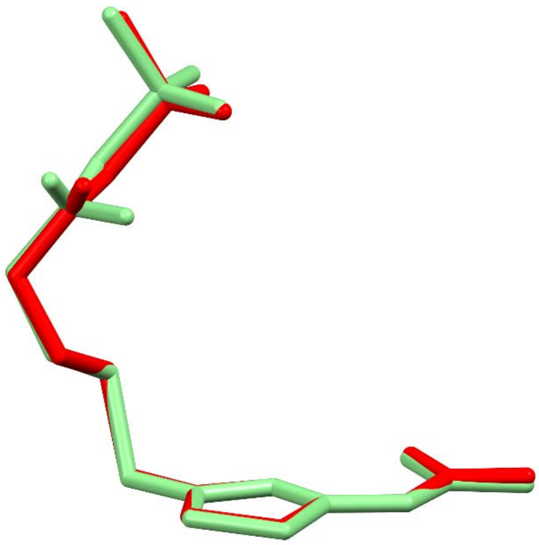

Figure S3 Overlay of the two symmetry-independent famotidine molecules in complex (**II**).

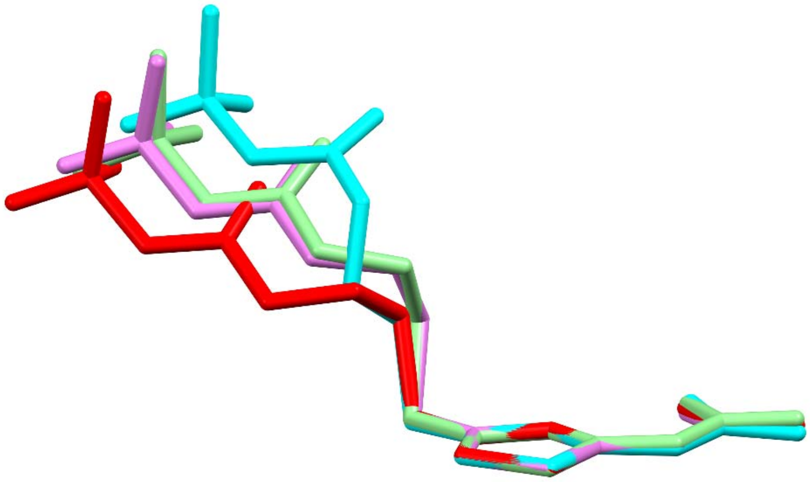

Figure S4 Overlay of the four crystallographically-independent molecules in complex (**IV**).
